# Quantitative Visualization of Console Behavior During Robot-Assisted Radical Prostatectomy Using the Da Vinci 5 System: A Case-Based Technical Report

**DOI:** 10.7759/cureus.105463

**Published:** 2026-03-18

**Authors:** Yuta Umezawa, Go Kaneko, Daisuke Igarashi, Suguru Shirotake, Masafumi Oyama

**Affiliations:** 1 Uro-Oncology, Saitama Medical University International Medical Center, Hidaka, JPN

**Keywords:** da vinci 5, force feedback, motion analysis, prostate cancer, robot assisted radical prostatectomy

## Abstract

Robot-assisted radical prostatectomy (RARP) using the da Vinci system (Intuitive Surgical, Inc., Sunnyvale, CA) has become a standard minimally invasive treatment for nonmetastatic prostate cancer. The next-generation da Vinci 5 system incorporates integrated motion analytics and force-feedback instruments, enabling quantitative visualization of console behavior during robotic surgery. In this case-based technical report, we describe our initial real-world experience with these functions in two patients who underwent RARP at a single institution. Both procedures were performed via a standard transperitoneal approach without pelvic lymph node dissection, and perioperative outcomes were acceptable in both cases. Digital logs obtained from the cloud-based My Intuitive platform (Intuitive Surgical) provided instrument-tip force categories, kinematic metrics including instrument and endoscope path lengths, and system events such as endoscope clutches, hand-controller clutches, and energy-pedal activations. The procedures were automatically segmented into three steps by the My Intuitive platform algorithm, and the analytics were reviewed in synchronization with the corresponding operative videos. Quantitative review demonstrated differing patterns of console motion, clutch use, and force application between the two analyzed procedures, particularly during dissection between the rectum and prostate and during vesicourethral anastomosis. Case 1 showed more frequent clutch use and a broader working range, whereas Case 2 showed a higher proportion of time categorized as force ≥ 6.5 N despite similar mean forces. This report demonstrates the technical feasibility of capturing, synchronizing, and visualizing console behavior using the integrated analytics of the da Vinci 5 system during RARP. Although the findings are descriptive and hypothesis-generating only, this integrated review approach may have potential value for structured technical reflection, data-guided feedback, and future surgical education research.

## Introduction

Robot-assisted radical prostatectomy (RARP) for nonmetastatic prostate cancer provides oncological outcomes equivalent to open or laparoscopic surgery, while reducing blood loss, shortening hospital stay, and promoting faster recovery of continence and sexual function [1, 2]. RARP has largely replaced open and laparoscopic radical prostatectomy and has become a widely adopted minimally invasive approach worldwide [3].

As robotic platforms continue to evolve, increasing attention has been directed not only toward perioperative outcomes but also toward objective assessment of surgical performance, technical efficiency, and console behavior during robotic procedures. The da Vinci 5 system (Intuitive Surgical, Inc., Sunnyvale, CA) represents the latest generation of the da Vinci multi-port robotic platform. It offers an enhanced console, improved visualization, digital features, and force-feedback instruments that measure and transmit instrument-tip forces to the surgeon [4].

In addition to real-time feedback, the associated cloud-based My Intuitive platform (Intuitive Surgical, Inc.) records system-derived quantitative data such as instrument-tip force categories, instrument and endoscope path lengths, and console events including clutch use and energy-pedal activations. These data can be reviewed together with synchronized operative video, potentially allowing step-specific evaluation of intraoperative console behavior. Although such functions may have future applications in surgical education, coaching, and performance science, their clinical interpretation remains incompletely defined.

This case-based technical report describes the quantification and visualization of console behavior using digital console logs from two RARP procedures performed with the da Vinci 5 system at our institution. The primary aim was to illustrate the technical feasibility and potential educational relevance of these integrated analytics in actual clinical practice, rather than to formally compare surgeon performance.

## Case presentation

The present report does not describe an unusual symptom-based presentation or a rare disease entity. Rather, it presents two real-world cases of nonmetastatic prostate cancer treated with RARP, selected to illustrate how the integrated motion and force analytics of the da Vinci 5 system can be reviewed in actual surgical practice.

Both patients underwent standard preoperative evaluation, including serum prostate-specific antigen measurement, imaging assessment, and histopathological diagnosis of prostate adenocarcinoma, before surgical treatment was selected. Both procedures were performed at the Saitama Medical University International Medical Center in Hidaka, Japan. Table 1 summarizes patient characteristics, surgical outcomes, and perioperative data. Case 1 underwent bilateral nerve-sparing RARP performed by a certified proctor accredited by the Japanese Urological Association and the Japanese Society of Endourology and Robotics. Case 2 was performed by a non-proctor surgeon with 24 prior RARP cases. These operator backgrounds are provided for procedural context only and are not intended for comparative inference. Both procedures were conducted using a standard transperitoneal approach without pelvic lymph node dissection. Videos 1-4 are operative video clips from the reported cases and correspond to the operative scenes reviewed in synchronization with My Intuitive analytics.

**Table 1:** Patient characteristics and perioperative outcomes of the two RARP cases performed with the da Vinci 5 system BMI: body mass index; PSA: prostate-specific antigen; EBL: estimated blood loss

Parameters	Case 1	Case 2
Patient characteristics		
Age (years)	63	73
BMI (kg/m2)	25.6	18.7
Prostate volume (mL)	22.1	50.3
PSA (ng/mL)	7.2	13.9
Gleason score	3+4	3+4
Clinical stage	cT1cN0M0	cT3aN0M0
Surgical outcomes		
Console time (min)	166	162
EBL (mL)	15	250
Postoperative outcomes		
Duration of urethral catheterization (days)	6	7
Length of postoperative hospital stay (days)	6	8

Digital console logs were collected using My Intuitive, a cloud-based analytics application that automatically records da Vinci system data. The recorded metrics included instrument-tip force categories, numbers of endoscope clutches, hand-controller clutches, and energy-pedal activations, as well as instrument and endoscope path lengths (Table 2).

**Table 2:** Digital console metrics recorded by the da Vinci 5 system during RARP, showing movement and force parameters for each case across platform-generated procedural steps The My Intuitive platform automatically segmented each procedure into three parts, which were labeled by the authors for descriptive review as follows: Step 1, exposure and endopelvic fascia incision; Step 2, bladder neck dissection to urethral transection; and Step 3, reconstruction phase. “-” indicates not applicable or not available for that step in the platform output. Force metrics are presented separately by instrument type because the Maryland bipolar was used mainly during the dissection phases, whereas the needle driver was relevant during the reconstructive phase. The ≥ 6.5 N category represents the upper force category displayed by the My Intuitive platform and should not be interpreted as a clinically validated safety threshold. All console metrics are presented descriptively and should not be interpreted as standalone validated measures of surgical quality.

Parameter		Overall		Step 1		Step 2		Step 3	
		Case 1	Case 2	Case 1	Case 2	Case 1	Case 2	Case 1	Case 2
Console time (min)		166.1	162	34.5	44.1	74.1	61	57.5	56.8
Endoscope clutch count		1284	724	163	210	481	255	638	255
Hand controller clutch count		810	320	184	166	326	120	300	34
Energy pedal count		769	521	350	203	409	281	7	30
Total instrument path length (m)		242.9	233.9	60.5	68	90.2	86.1	92.2	79.8
Force (Maryland bipolar)									
Average (N)		2.1	1.9	1.9	1.8	1.5	1.4	-	-
Time at ≥ 6.5 N (%)		2.2	7.7	0.8	1.9	0	3.5	-	-
Force (Needle driver)									
Average (N)		1.5	1.8	-	-	-	-	1.4	1.9
Time at ≥ 6.5 N (%)		2.1	7.1	-	-	-	-	2.3	7.6

**Figure 1:**
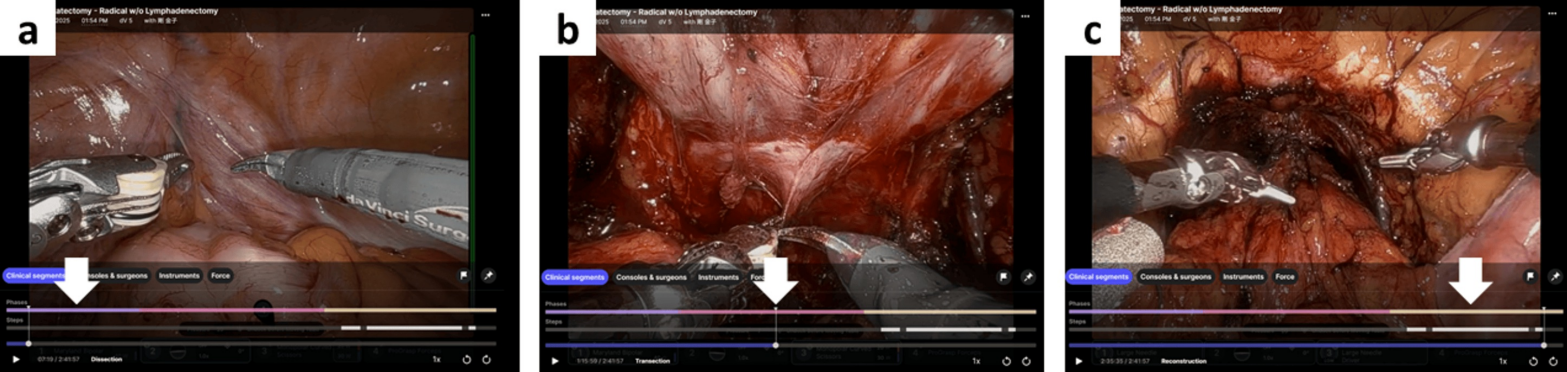
Automatic step segmentation of RARP in the My Intuitive platform Each procedure was automatically segmented into three parts by the cloud-based My Intuitive algorithm, which displays both operative video and corresponding analytic data. For descriptive review, these parts were labeled by the authors as follows: (a) Step 1, from console start to exposure and endopelvic fascia incision (purple bar, white arrow); (b) Step 2, bladder neck dissection to urethral transection (pink bar, white arrow); and (c) Step 3, reconstruction phase (yellow bar, white arrow). These labels were assigned for interpretive clarity and do not necessarily represent a universally standardized classification of RARP phases.

Force-related metrics were summarized separately by instrument type because the Maryland bipolar was primarily used during the dissection phases, whereas the needle driver was relevant during the reconstructive phase. The My Intuitive platform algorithm automatically segmented each operation into three parts and synchronized the analytics with the corresponding operative video (Figure 1). For descriptive step-specific review, these parts were labeled by the authors as follows: Step 1, exposure and endopelvic fascia incision; Step 2, bladder neck dissection to urethral transection; and Step 3, reconstruction phase. These labels were assigned for interpretive clarity and do not necessarily represent a universally standardized classification of RARP phases.

**Figure 2:**
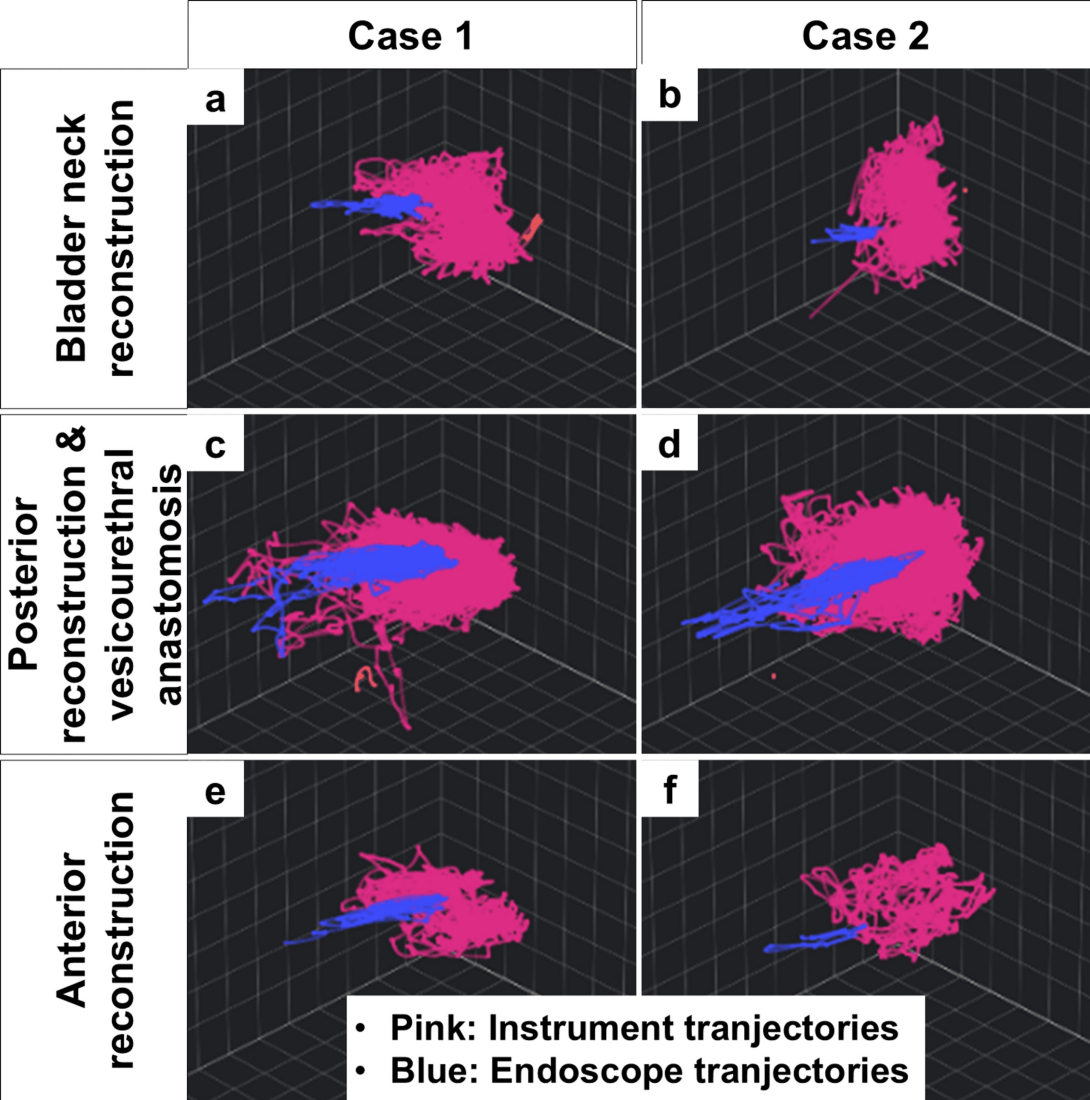
Three-dimensional trajectories of instruments and endoscope during Step 3 The left column shows Case 1 and the right column shows Case 2. Pink lines represent instrument trajectories and blue lines represent endoscope trajectories. Within the My Intuitive platform, Step 3 was automatically divided into three parts. For descriptive review, these parts were labeled by the authors as bladder neck reconstruction, posterior reconstruction with vesicourethral anastomosis, and anterior reconstruction. The trajectory displays are presented descriptively to illustrate differences in working range and movement patterns between the two analyzed procedures.

Quantitative review showed differences between the two analyzed procedures in the numbers of endoscope clutches, hand-controller clutches, and energy-pedal activations, with higher counts observed in Case 1, particularly in Steps 2 and 3 (Table 2). Visualization of instrument and endoscope trajectories during Step 3 also suggested a broader working range in Case 1 (Figure 2). On synchronized video review, the procedure in Case 1 showed more frequent movement of the instruments and endoscope across a wider pelvic field during needle exchange and suture handling, whereas the working field in Case 2 appeared relatively narrower. These findings are presented descriptively as examples of how variation in console behavior can be visualized in actual RARP procedures and should not be interpreted as evidence of superior or inferior technical performance.

At instrument-tip forces exceeding 6.5 N, the My Intuitive platform displayed “≥ 6.5 N” rather than the exact value. Because 6.5 N represents the upper limit of the force category reported by the system, we operationally summarized any period with a recorded force of ≥ 6.5 N as a “high-force episode” for descriptive purposes only. This threshold does not represent a clinically validated cutoff for tissue injury or unsafe manipulation. Although the average left-hand instrument forces were similar between the two procedures, the proportion of time with force ≥ 6.5 N was higher in Case 2 (Table 2). In Step 2, no force ≥ 6.5 N was recorded in Case 1, whereas force ≥ 6.5 N was observed during 3.5% of the step duration in Case 2. Synchronized review of the operative videos during dissection between the rectum and prostate showed that the procedure in Case 1 involved a combination of blunt and sharp dissection (Video 1), whereas blunt dissection predominated in Case 2 and episodes categorized as ≥ 6.5 N were observed more frequently (Video 2).

**Video 1:** Dissection between the rectum and prostate in Case 1

**Video 2:** Dissection between the rectum and prostate in Case 2

A review of the Step 3 videos also showed more frequent instances in Case 2 in which greater traction appeared to be applied to the suture with the left-hand needle driver (Videos 3-4).

**Video 3:** Suture reeling during vesicourethral anastomosis in Case 1

**Video 4:** Suture reeling during vesicourethral anastomosis in Case 2

However, the clinical implications of these force-related observations remain uncertain, and the differences observed in this report may also have been influenced by case complexity, anatomy, and procedural context.

## Discussion

In these two cases, the quantitative analytics embedded in the da Vinci 5 system enabled objective visualization of console motion and force application during RARP. The two procedures showed different patterns of clutch use, movement range, and force-category distribution, demonstrating that the integrated platform can capture and display measurable variation in console behavior. However, because each surgeon contributed only a single procedure, these observations should be regarded strictly as descriptive and illustrative rather than as evidence of systematic differences in surgical skill, performance, or superiority.

A central purpose of this report is not comparative evaluation, but technical demonstration. The findings should therefore be interpreted as showing the feasibility of synchronizing console-derived analytics with operative video in real-world RARP. This platform-based review may support structured postoperative reflection by allowing surgeons to examine motion efficiency, working range, and force-related events in relation to specific operative steps. At the same time, the present report does not establish validated performance thresholds, define optimal patterns of console behavior, or demonstrate that any measured metric directly reflects surgical quality.

Several console-derived metrics used in this study may require contextual explanation. Endoscope and hand-controller clutch counts may reflect repositioning behavior and changes in working range during different phases of the operation. Instrument and endoscope path lengths may provide a descriptive representation of how extensively the instruments move within the operative field. Energy-pedal activations may reflect the frequency of electrosurgical device use during specific tasks. In the present report, these metrics were included to illustrate how the da Vinci 5 analytics platform captures multiple dimensions of console activity. However, they should not be interpreted as standalone validated indicators of surgical efficiency or quality.

The interpretation of force-related parameters warrants particular caution. Although the mean applied forces were similar, Case 2 included more episodes in the “≥ 6.5 N” category than Case 1. Importantly, the threshold of 6.5 N used in this study was not selected as an empirically validated safety cutoff. Rather, it reflects the reporting format of the My Intuitive platform, which aggregates all higher force values into a single “≥ 6.5 N” category. In this context, Step 2 was informative because no events in this category were observed in Case 1, whereas such events were recorded in Case 2. However, any suggestion that these differences reflect safer tissue handling, more refined technique, or lower risk of tissue injury remains speculative and should not be inferred from these two cases alone. Accordingly, the principal significance of these observations is descriptive rather than evaluative.

Previous motion-analysis studies using earlier-generation robotic systems have shown that automated kinematic metrics can distinguish between levels of surgical experience and may correlate with postoperative outcomes. Hung et al. reported that performance metrics derived from instrument kinematics and system events could quantify surgical skill and, when combined with clinicopathological variables, predict early continence recovery after RARP [5-7]. Recent systematic reviews have further highlighted the growing role of objective tools, surgical data analytics, and artificial intelligence in technical skill assessment in robotic and minimally invasive surgery, while also emphasizing the need for further validation, standardization, and demonstration of clinical relevance [8, 9]. In this context, integrated platform-based analytics may provide a practical foundation for future data-guided technical review and educational feedback. Our findings support the educational relevance of motion analysis. However, we found that such work has largely been limited to specialized centers because prior robotic platforms did not routinely integrate analytics into everyday clinical workflows. By contrast, the da Vinci 5 system incorporates motion-analysis tools directly into the platform, potentially enabling more practical and scalable performance review in routine robotic surgery.

Force control represents a complementary dimension of surgical assessment. The lack of tactile feedback has long been recognized as a limitation of robotic surgery because excessive or uncontrolled traction may contribute to tissue injury. Newer platforms, such as Senhance and Saroa, address this issue by providing tactile or force feedback to help surgeons perceive tissue resistance and avoid excessive grasping [10-13]. The da Vinci 5 system is the first widely adopted multi-port platform to integrate force-feedback instruments that measure and transmit real-time forces to the surgeon. Early reports have suggested that force feedback may reduce traction force during robotic procedures by as much as 40% [4]. In our experience, these features appear to enhance awareness of traction and suture tension, while postoperative review of quantified force data may facilitate reflective learning. Nevertheless, the educational and clinical benefits of these functions remain to be established in larger studies.

This case-based technical report has several important limitations. First, it includes only two procedures performed by two different surgeons and is therefore inherently underpowered for any statistical inference or comparative interpretation. The present observations should not be regarded as evidence that the identified motion or force patterns reflect reproducible differences in surgeon performance. Second, the two patients differed in baseline characteristics, including age, body mass index, clinical stage, and particularly prostate volume (22.1 mL vs. 50.3 mL), all of which may have influenced exposure, working space, traction requirements, dissection strategy, and console behavior independently of surgeon experience. Third, our analysis relied exclusively on console-derived metrics from the My Intuitive platform. Because this system reports all forces above 6.5 N within a single “≥ 6.5 N” category, force intensity above that threshold could not be further differentiated. In addition, these metrics do not capture all determinants of surgical quality, such as tissue fragility, bleeding tendency, assistant performance, or overall team dynamics. Fourth, the present report does not correlate console analytics with important clinical outcomes such as positive surgical margins, continence recovery, erectile function, or postoperative complications. Finally, this was a single-institution early experience with the da Vinci 5 system, and the generalizability of the findings to other surgeons, institutions, and procedures remains uncertain.

Future studies involving larger numbers of surgeons and procedures, ideally with synchronization of console analytics and surgical video, will be necessary to determine whether step-specific motion and force patterns are reproducible, educationally actionable, and clinically meaningful. Correlation with perioperative, oncologic, and functional outcomes will also be important to establish the practical relevance of these analytics. Such approaches may ultimately support a more data-driven framework for RARP training, reflective learning, and technical feedback. At present, our findings should be viewed as an initial, hypothesis-generating demonstration of how the da Vinci 5 system can visualize console behavior in a clinically feasible manner.

In summary, this report should be interpreted as a descriptive, case-based technical demonstration rather than as comparative evidence of surgeon performance or a formal learning-curve analysis. The integrated motion and force analytics of the da Vinci 5 system enabled visualization of console behavior in two real-world RARP cases and may offer educational value by supporting structured review and feedback. Although the present findings are limited by the small number of cases and their descriptive nature, they suggest the potential utility of these metrics as training-support tools. Further investigation in larger cohorts is needed to determine their reproducibility, validity, and broader role in surgical education and data-guided technical feedback.

## Conclusions

These two cases illustrate the technical feasibility of visualizing motion and force parameters using the integrated analytics of the da Vinci 5 system during RARP. Although descriptive and limited in scope, this report suggests the potential educational utility of these metrics for structured review of console behavior. Larger studies are needed to determine whether these analytics correlate with surgeon skill, learning curves, and perioperative, oncologic, or functional outcomes.
